# Stress Concentration Factors for Welded Plate T-Joints Subjected to Tensile, Bending and Shearing Loads

**DOI:** 10.3390/ma14030546

**Published:** 2021-01-24

**Authors:** Krzysztof L. Molski, Piotr Tarasiuk

**Affiliations:** Faculty of Mechanical Engineering, Bialystok University of Technology, Wiejska 45C, 15-351 Bialystok, Poland; k.molski@pb.edu.pl

**Keywords:** welded plate T-joint, stress concentration factor, weld toe, finite element analysis, axial, bending and shearing load

## Abstract

The paper deals with the problem of stress concentration at the weld toe of a plate T-joint subjected to axial, bending, and shearing loading modes. Theoretical stress concentration factors were obtained from numerical simulations using the finite element method for several thousand geometrical cases, where five of the most important geometrical parameters of the joint were considered to be independent variables. For each loading mode—axial, bending, and shearing—highly accurate closed form parametric expression has been derived with a maximum percentage error lower than 2% with respect to the numerical values. Validity of each approximating formula covers the range of dimensional proportions of welded plate T-joints used in engineering applications. Two limiting cases are also included in the solutions—when the weld toe radius tends to zero and the main plate thickness becomes infinite.

## 1. Introduction

Welded T-connections are commonly used engineering structures. Their significant advantage lies in low costs of production due to the use of simple elements, like tubes of circular or rectangular cross sections and plates, usually connected by welding. There are many types of such joints produced in various configurations: as tubular, tubular-plate, and plate couples. The fatigue failure of welded structural components subjected to cyclic loads is the most frequent cause of damage, which begins at critical zones where stress concentration occurs [1]. Various standards and recommendations presented in References [2,3,4,5,6,7] are very helpful in designing welded structures.

Stress concentration factor (SCF) values determined for the known geometrical and loading conditions are of great importance in fatigue design because they enable relating the loading history to the critical zone of the structure. Some examples of fatigue analysis in tubular joints applied to various types of structures and devices are given in References [8,9,10,11,12] where stress concentration plays a key role. Fatigue damage analysis considering crack initiation and propagation processes in welded T-joints was published in References [13,14,15]. Numerous formulas of stress concentration factors for various types of welded T-joints, based on the finite and boundary element analyses, were presented in References [16,17,18,19,20].

Some important phenomena associated with welding processes consist of a random character of key geometrical parameters influencing the SCF value. In such cases, appropriate statistic data obtained directly from real structures and regarding changed values and shapes of weldments are necessary. Examples of such data in the form of histograms can be found in the literature [21,22,23,24,25]. Various types of the weld face geometry—plane, convex, or concave—may be obtained during manufacturing, which generally depend on the welding method, position, and parameters of the process, e.g., welding speed, current, etc. Some examples of real weld shapes and profiles influencing SCF values in cases of as-welded and intentionally improved weld toe zones are also given in References [25,26,27,28,29]. Many techniques have also been developed to improve the endurance limit and reliability of the structure (grinding, milling, re-melting techniques, etc.) [30]. Each process produces specific radii influencing local stress concentration.

The application of the known parametric formulas in fatigue design encounters some difficulties arising from their accuracy, range of validity, and different ways of defining SCF. On the other hand, assessment of fatigue life requires high accuracy of SCF solutions and should cover the whole range of values of all key parameters influencing SCFs.

The present study deals with determination of stress concentration factors for non-load carrying welded plate T-joints subjected to axial, bending and shearing loading modes. The main objective was to develop highly accurate SCF formulas of a wide ranging validity, including two limiting cases: when the toe radius *ρ* tends to zero and when the main plate thickness *t* becomes infinite.

## 2. Methodology

### 2.1. General Assumptions

**Figure 1:**
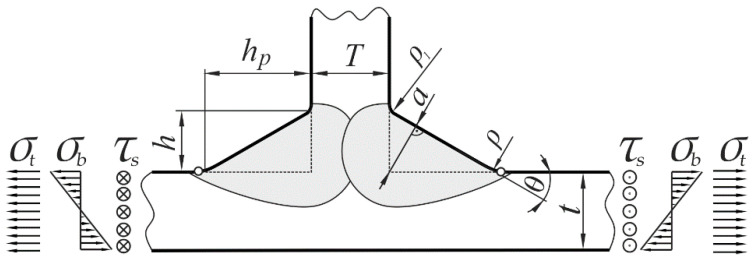
Geometrical parameters and loading modes of the plate T-joint.

Five key geometrical parameters—*ρ*, *a*, *θ*, *t,* and *T*—have been selected and considered as independent variables, where *t* and *T* represent the main and attachment plate thickness, respectively, *ρ* is the weld toe radius, *θ* indicates the weld angle, and *a* is the weld throat thickness defined here as the shortest distance between the joint root and the weld face. Leg length parameters, *h* and *h*_p_, characterizing the weld size, are also used in the literature, e.g., in References [16,17,18]. Shape and geometrical dimensions of a plate T-joint, as well as three loading modes considered in the present work, are depicted schematically in Figure 1. Small circles drawn symmetrically at the weld toe zone indicate locations of the maximum stresses.

The present analysis considered the following assumptions.

1. Joint material is linear elastic, isotropic, and homogeneous.
2. Elastic properties of the weld material and the base material, represented by elastic constants, are the same.
3. The welded joint is free from residual stresses, structural irregularities, and imperfections, including lack of penetration defects.
4. Both welds are symmetrical with respect to the axis of the attachment plate, which is perpendicular to the main plate.
5. Weld faces are plane and contour of the weldment is smooth with a transition toe radius *ρ* > 0.
6. External load – axial, bending, and shearing – is applied at the opposite sides of the main plate sufficiently far from the welds.
7. Small deformations occur in the whole body.
8. Five geometrical parameters: *ρ*, *a*, *θ*, *t*, and *T* are considered as independent variables varying in the following ranges: 0 < *ρ*/*a* ≤ 1.3, 0 < *a*/*t* ≤ 1.3, 1 ≤ *T*/*a* ≤ 4, and 30° ≤ *θ* ≤ 60°.
9. Stress concentration factors for axial, bending, and shearing loads are defined as *K_tt_* = σ_1max_/σ_t_, *K_tb_* = *σ*_1max_/*σ*_b_, and *K_ts_* = τ_max_/τ_s_, respectively.

Theoretical stress concentration factor SCF is valid for linear-elastic, homogeneous, and isotropic materials as well as bodies of defined geometry, loading and displacement boundary conditions. Any departure from these assumptions, as material anisotropy or geometrical irregularities (flaws, imperfections, etc.) may significantly change the stress distribution in the body, including the maximum stress value at the notch root.

The aim of the work was to obtain SCF approximating formulae covering possible proportions between geometrical parameters of the T-joint used in engineering practice. For this reason, the supposed margin for the normalized parameters was very wide. The weld toe radius *ρ* 1.3 times larger than the weld throat may be considered useless for practical applications, but such a limiting value can guarantee that all smaller radii fall into the range of application. The second case when *a*/*t*→0 may also take place in engineering applications while a relatively thin plate is welded to a very thick main plate.

### 2.2. General Approach

The procedure of reaching main objectives of the present study consisted of several steps. First, conveniently defined new geometrical parameters had to be specified. Due to the fact that SCF values do not vary when all dimensions of the body proportionally change, the following normalized non-dimensional quantities were introduced.

(1) $$X=\rho /\left(\rho +a\right)=\frac{\rho /a}{\rho /a+1}$$

(2) $$Y=a/\left(a+t\right)=\frac{a/t}{a/t+1}$$

(3) Z=T/a

In this way, four independent variables *X*, *Y*, *Z*, and *θ*, containing the five key dimensions of the joint, were taken into consideration. The upper weld toe radius, *ρ*_1_, shown in Figure 1, was chosen as equal to 0.1*a* and had no influence on stress concentration factors. The second step consisted of numerical modelling of the joint using the finite element method, where mutual proportions between the basic joint dimensions systematically changed. In this way, several thousands of SCF values, covering the assumed range of validity, were determined. More detailed information on the numerical modelling and SCF calculating procedures are presented in the next section. The third step of the approach consisted of choosing a general form of the mathematical representation of SCFs’ approximating functions. An expression applied in the present study was similar to that presented by the authors in Reference [31], where a cruciform joint of *θ* = 45°, subjected to tensile and bending loads, was analysed. However, the problem considered in the present work was more general than the previous one because the weld angle *θ* varied in the range of 30–60°, increasing the number of variables in the parametric formula. In the numerical Finite Element Method (FEM) modelling, the weld angle *θ* was changed by 2.5° and the whole calculating procedure, carried out for a given angle, had to be repeated 12 times. Hence, a general form of the approximating function is given by Equation (4).

(4) $$K_{t}=X^{n}P\left(X,Y,\theta ,Z_{0}\right)\kappa \left(X,Y,Z,\theta ,Z_{0}\right),$$

where a singular term *X^n^* accounts for the stress concentration effects when *ρ*→0. The function *P*(*X*,*Y*,*θ*,*Z*_0_), represented by polynomials, can be derived from numerical SCF solutions normalized with respect to the singular term, *K_t_/X^n^* for different *X*, *Y*, and *θ*, while *Z* = *Z*_0_. Equation (4) indicates that the *P* function cannot directly relate SCF to *Z* because it is derived for a particular, arbitrarily chosen value of *Z* = *Z*_0_. For this reason, the additional correction function is *κ*(*X*,*Y*,*Z*,*θ*,*Z*_0_), satisfying Equation (5),

(5) $$\kappa \left(X,Y,Z=Z_{0},\theta \right)=1$$

was necessary to account for the relative stiffener thickness on SCF. In other words, the function *κ* can be interpreted as a multiplier, which enables us to calculate SCF for any value of *Z* with respect to the one corresponding to *Z*_0_, for the known parameters *X*, *Y*, and *θ*.

The exponent *n*, denoted as *n*_s_ for shearing load, is necessary to describe stress concentration effects when the weld toe radius tends to zero. In References [31,32], it was proven that the correct *n* values are the same as the stress field singularity exponents corresponding to the characteristic angle of the sharp corner [33], into which the weld toe region is converted while the weld toe radius *ρ* tends to zero. According to Reference [32], numerical values of the exponent *n* can be calculated for axial and bending load, using Equation (6),

(6) $$n=\frac{-0.63662\theta -0.09330\theta^{2}}{1+0.77635\theta +0.04075\theta^{1.5}-0.00499\theta^{2}+0.13365\theta^{2.5}}$$

which is valid in the range of 0 ≤ *θ* ≤ π/2 with an accuracy of five significant digits.

For anti-plane deformation produced by the shearing load, the exact value of the exponent *n*_s_ equals:

(7) $$n_{s}=\frac{-\theta }{\theta +\pi }$$

where *θ* is in radians.

The next step of the procedure consisted of approximating the *P*(*X*,*Y*,*Z*_0_,*θ*) function to the normalized numerical SCF data using the least squares method. After particular constants (coefficients and exponents) of the *P* function were determined, the approximation accuracy was verified. In this way, one part of the solution, where *Z* = *Z*_0_, was completed. The final step of the present approach consisted of finding unknown coefficients and exponents of the correction function *κ*(*X*,*Y*,*Z*,*θ*,*Z*_0_) accounting for the influence of *Z* values on SCFs. After performing the second validation of the full solution including *κ*, the close form approximating functions for calculating SCFs were determined. The whole procedure was consecutively repeated for each loading mode.

## 3. Numerical FEM Modelling and Some SCFs’ Results

### 3.1. Tensile and Bending Load

**Figure 2:**
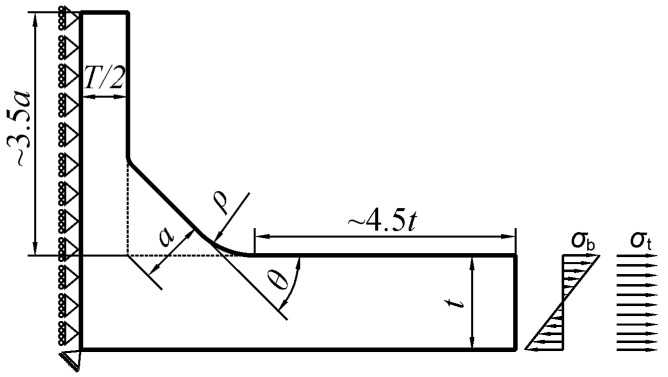
Basic geometrical parameters and boundary conditions of the numerical FEM model for tensile and bending loads.

Systematic numerical modelling of plate T-joints was carried out by using the Finite Element Method (FEM) incorporated in the ANSYS 19 Multiphysics program. The PLANE182 finite elements that were used were defined by four nodes having 2 degrees of freedom at each node. The elastic material constants were assumed to be similar to those for steel, where Young’s modulus E = 210 GPa and Poisson’s ratio ν = 0.3. However, according to the theory of elasticity, stress field in the body and SCF values do not depend on the elastic constants of the material in all cases modeled. Loading and displacement boundary conditions imposed on one half of the body, as well as its shape, are shown in Figure 2. According to the principle of de Saint Venant, the minimum length of the main plate measured from the weld toe was about 4.5*t*. This value was confirmed by conducting preliminary numerical tests.

**Figure 3:**
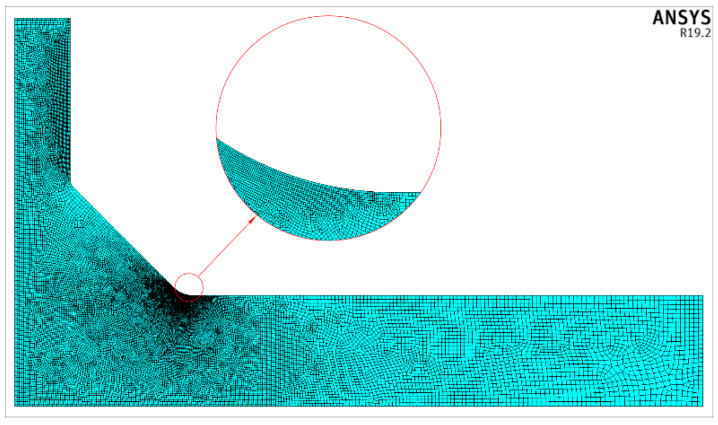
Example of a finite element mesh for *θ* = 45°, while *X* = 0.25, *Y* = 0.415, and *T*/*t* = 1.

Approximately 820,000 finite elements were used for each geometrical case of the modelled T-joint. Special attention was paid to the finite element mesh density at the weld toe zone, where high values of the stress gradients occur. For this reason, the dimensional ratio of neighbouring finite elements was about 1.2 and approximately 40 to 70 finite elements were used along the weld toe circular arc, as described by the radius *ρ*. One example of a finite element mesh is shown in Figure 3.

**Figure 4:**
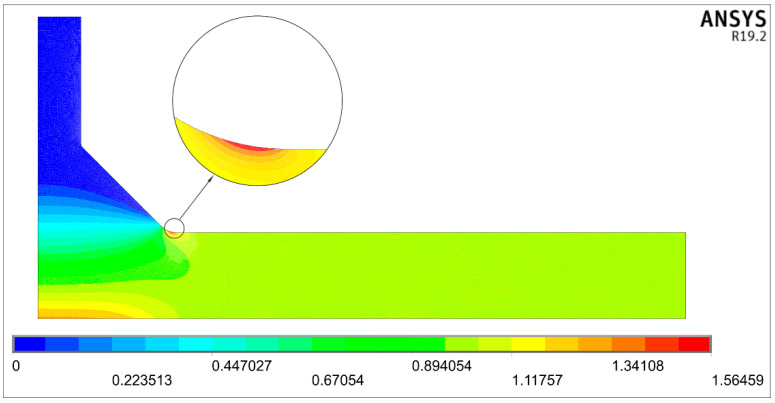
Distribution of the first principal stress *σ*_1_ for *σ*_t_ = 1 MPa, while *θ* = 45°, *X* = 0.25, *Y* = 0.415, and *T*/*t* = 1.

An example of the solution obtained for the tensile load for the nominal uniform stress *σ*_t_ = 1 MPa applied over the right end of the body is shown in Figure 4. The first principal stress results offer the most convenient representation because the particular value of the SCF is directly accessible. Therefore, the nominal stress in all numerical models was always equal to one.

In the presented case, the maximum principal stress σ_1max_ equals 1.56459 MPa, which is directly seen on the scale below the picture. This value is also interpreted as the stress concentration factor *K_tt_*.

### 3.2. Shearing Load

The formulation of two-dimensional problems for the anti-plane state of deformation is different than for in-plane loading modes. In the case of the anti-plane problem, only one displacement coordinate *W* in each point is sufficient to describe deformation of the body and *W(x,y)* may be treated as a scalar potential function *ψ(x,y)*. Therefore, a problem in an anti-plane state of deformation may be considered as a boundary value problem governed by Laplace’s equation, represented in Cartesian coordinates by Equation (8).

(8) $$\frac{\partial^{2}\Psi }{\partial x^{2}}+\frac{\partial^{2}\Psi }{\partial y^{2}}=0,$$

which is valid in the whole body and satisfies boundary conditions along the surrounding contour. The fact that the same relationship also holds for the temperature field *T*_temp_*(x,y)* in plane steady-state heat conduction problems leads to the conclusion that thermal analogy may be used to obtain solutions of stress concentration factors for an anti–plane state of deformation. A more detailed description of this approach can be found in Reference [32] where SCFs for butt-welded plates subjected to shearing load were determined using FEM.

**Figure 5:**
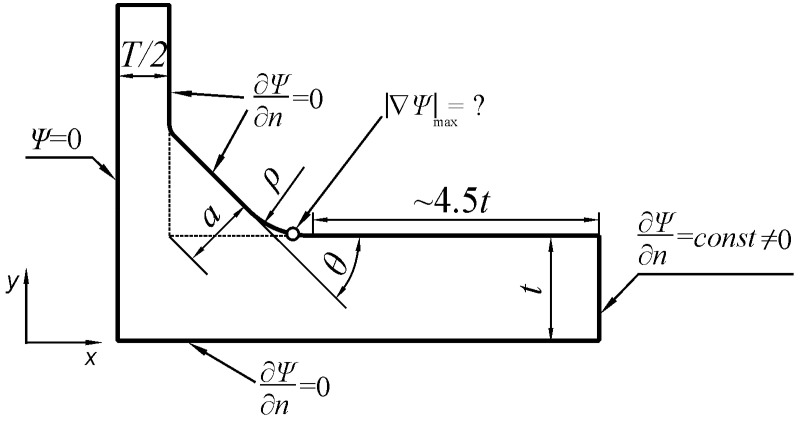
Shape and boundary conditions of the numerical FEM model for shearing load.

In the present analysis, the ANSYS 19 Multiphysics program with a Thermal module and PLANE55 finite element was used. A PLANE55 finite element is defined by four nodes with a single degree of freedom corresponding to the temperature at each node. Meshing of the modelled area was the same as in the previous cases for tensile and bending loads for identical joint geometry. The shape of the modelled body as well as mixed boundary conditions are shown in Figure 5.

Nominal uniform heat flux *q*_nom_ = 1 W/m^2^ was applied over the right-hand edge of the body, while zero temperature was applied to the left end along the plane of symmetry of the joint. Since the upper face and the lower face of the joint are free from external shearing loads, they have to be insulated in the thermal model. It is clear that the shear stress components, *τ_xz_* and *τ_yz_*, given by Equation (9):

(9) $$\tau_{xz}=G\frac{\partial W}{\partial x};\;\tau_{yz}=G\frac{\partial W}{\partial y},$$

and related to the partial derivatives of the potential function *W(x,y)* in particular directions, are proportional to the corresponding heat flux components, *q_x_* and *q_y_*, represented by Equation (10).

(10) $$q_{x}=-k\frac{\partial T_{\mathrm{temp}}}{\partial x};\;q_{y}=-k\frac{\partial T_{\mathrm{temp}}}{\partial y}.$$

Numerical SCF values were calculated as a ratio of the maximum magnitude of the temperature gradient |∇*T*_temp_|_max_ at the weld toe zone, shown by a small circle in Figure 5, to the magnitude of the nominal temperature gradient |∇*T*_temp_|_nom_ over the right side of the body. It is well known that SCF values in such cases do not depend on the conductivity *k* of the medium. Therefore, the same result is obtained by comparing corresponding heat flux quantities *q*_max_/*q*_nom_. In order to simplify relations between the heat flux and the magnitude of the temperature gradient, it was convenient to take *k* = 1 W/(Km).

**Figure 6:**
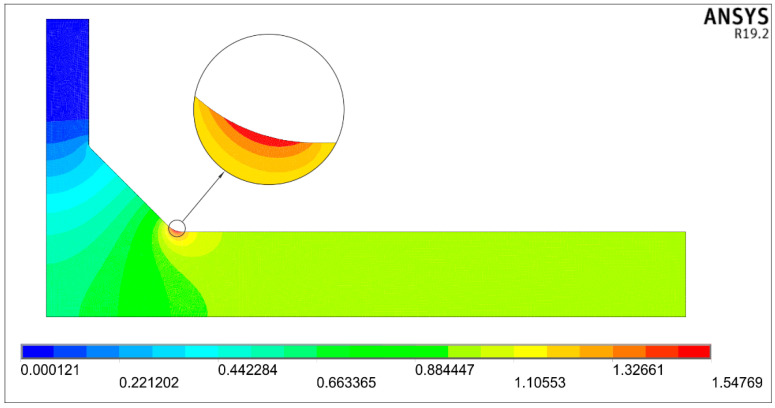
Heat flux *q* distribution, interpreted as shearing stress *τ* for *τ*_s_ = 1 MPa, while *θ* = 45°, *X* = 0.25, *Y* = 0.415 and *T*/*t* = 1.

One example of a steady-state heat conduction solution is shown in Figure 6 for the nominal heat flux *q*_nom_ = 1 W/m^2^ applied over the right edge of the main plate. Magnitudes of the flux *q*, obtained at each point of the body, make it possible to find directly *K_ts_* value equal 1.54769, which is shown on the scale below the picture. 

The maximum temperature corresponding to the unit nominal heat flux applied along the right end of the body was equal to 7.089 K. This temperature value resulted from the numerical solution of the boundary-value problem with mixed boundary conditions and was not directly applied over the right edge as a boundary condition.

In subsequent models of T-joints, particular values of *X*, *Y,* and *θ* were systematically changed, while the relative stiffener thickness *T*/*a* was constant and equal to one (*Z*_0_ = 1). One example of such a set of SCF results for a T-joint subjected to shearing load for *θ* = 45° is presented in Table 1.

Similar 12 sets of SCFs’ data were obtained for other weld angles *θ* in the range of 30–60° with a step of 2.5°. In this way, 3328 SCF numerical solutions were obtained for each loading mode, which means that 9984 cases of different T-joint shapes had to be modelled and solved for all loading modes.

## 4. SCF Approximation Formulas

### 4.1. Numerical and Graphical Representation of P Functions

**Figure 7:**
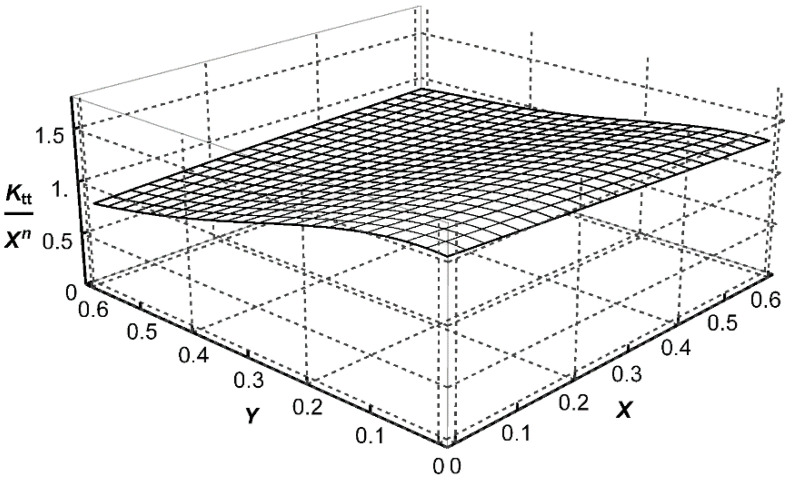
Graphical representation of the function $P_{t}=K_{tt}/X^{n}$ for *θ* = 45°, *n* = −0.3264 and *Z*_0_ = 1.

**Figure 8:**
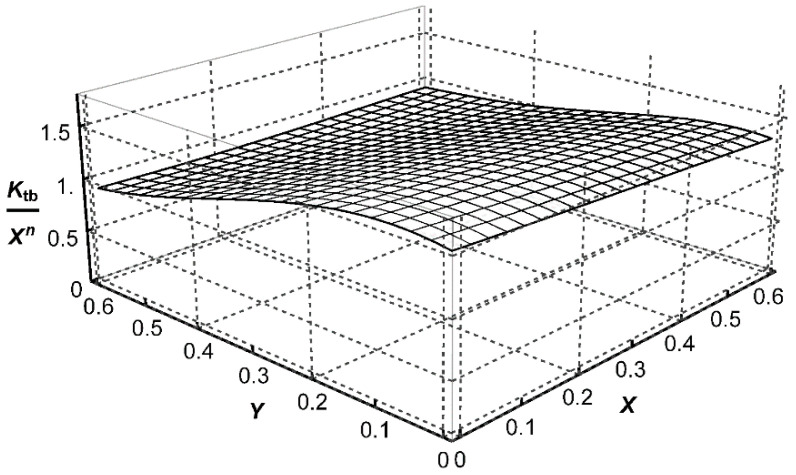
Graphical representation of the function $P_{b}=K_{tb}/X^{n}$ for *θ* = 45°, *n* = −0.3264 and *Z*_0_ = 1.

**Figure 9:**
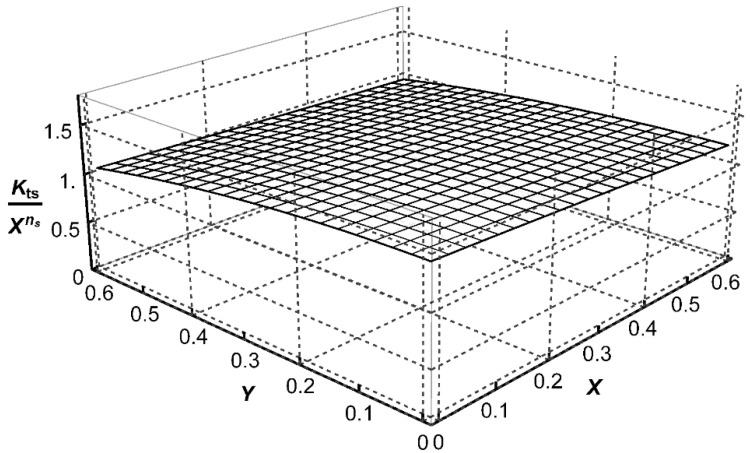
Graphical representation of the function $P_{s}=K_{ts}/X^{n_{s}}$ for *θ* = 45°, *n*_s_ = −0.2 and *Z*_0_ = 1.

After normalizing each SCF value with respect to a proper singular term $X^{n}$ , three approximating functions *P*_t_, *P*_b_, and *P*_s_, corresponding to each loading mode, were determined in the form of polynomials. Their mathematical representation is shown in brackets of Equations (A1)–(A3) given in Appendix A. Graphical representations of these functions, corresponding to the weld angle *θ* = 45° for three loading modes, are shown in Figure 7, Figure 8 and Figure 9. Numerical values of the exponents *n* = −0.3264 and *n*_s_ = −0.2 were obtained from Equations (6) and (7), respectively, for *θ* = π/4.

### 4.2. Validation of the P Functions

Accuracy of the approximating formulas of *P* functions was verified by comparing calculated SCFs values to their numerical equivalencies obtained using the finite element method. General formula, expressed by Equation (4), is now reduced to Equation (11).

(11) $$K_{t}=X^{n}P\left(X,Y,\theta ,Z_{0}\right),$$

where *Z* = *Z*_0_ = 1 and *κ* = 1 in the present case. Three examples of such comparisons are presented in Table 2, Table 3 and Table 4 for T-joints subjected to tensile, bending, and shearing loads, respectively, for *θ* = 45°.

Similar comparisons were made for various weld angles *θ* in the range of 30–60°. The maximum percentage error of the approximation was lower than 2%.

### 4.3. Determination of the Correction Functions κ

Additional FEM modelling was carried out in order to determine the influence of the relative attachment plate thickness *Z* = *T*/*a* on SCF, represented mathematically by a correction function *κ*. Several thousand new cases of a T-joint were modelled and solved for each loading mode and various proportions of *T*/*a* in the range of 1 ≤ *T*/*a* ≤ 4.

**Figure 10:**
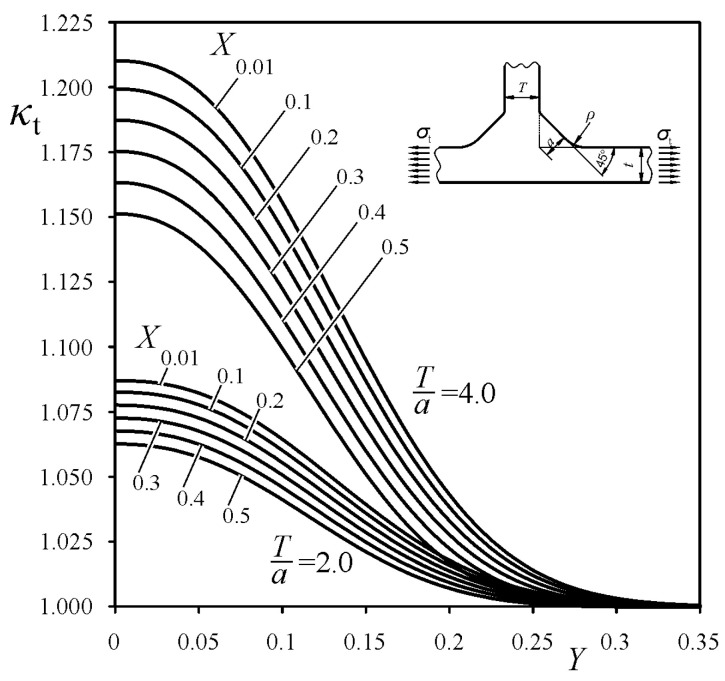
Correction function *κ*_t_ for tensile load, while *θ* = 45°.

**Figure 11:**
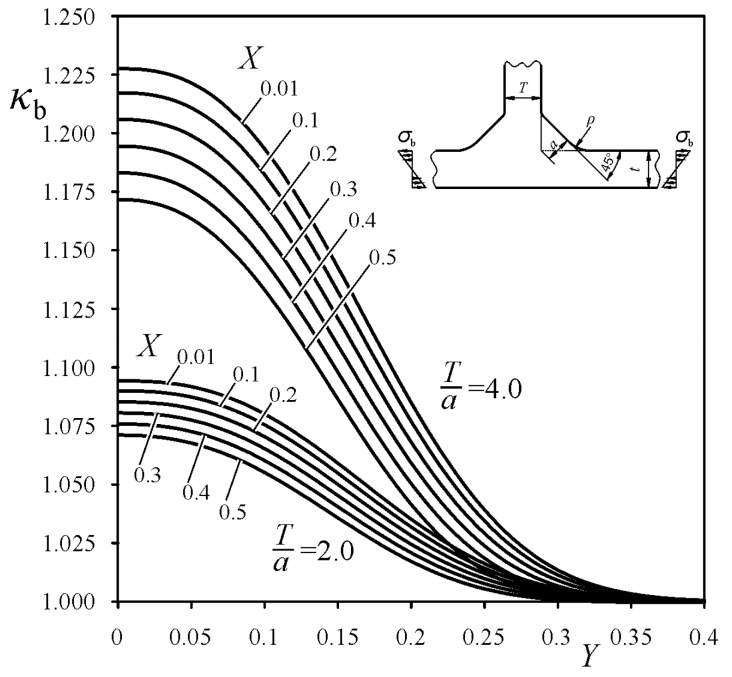
Correction function *κ*_b_ for the bending load while *θ* = 45°.

**Figure 12:**
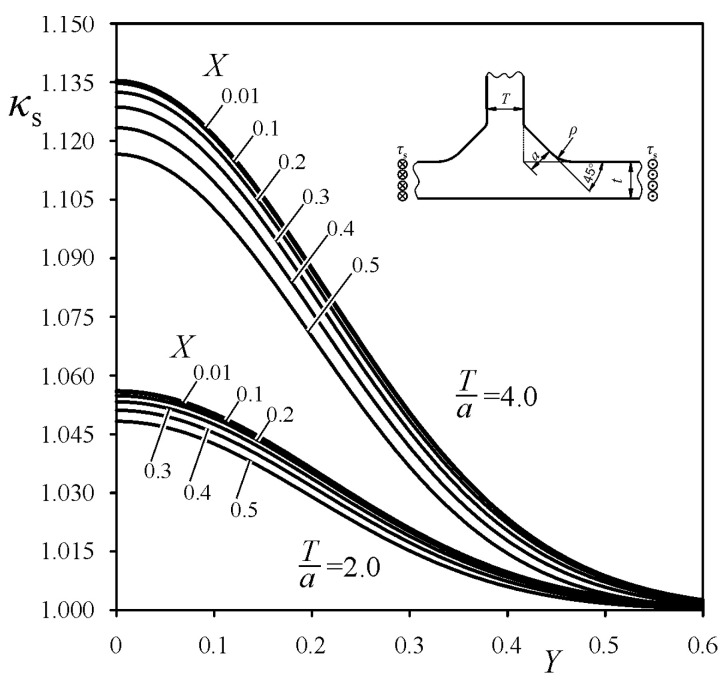
Correction function *κ*_s_ for the shearing load while *θ* = 45°.

The general mathematical representation of the correction functions *κ* in the form of Equation (12).

(12) $$\kappa \left(X,Y,Z,\theta ,Z_{0}\right)=1+\left(\sqrt{Z}-\sqrt{Z_{0}}\right)\left[1-\left(B_{1}+B_{2}Y^{2}\right)X^{m}\right]Exp\left[-{\left(B_{3}Y\right)}^{p}-B_{4}\right]$$

was derived, which is similar to the one performed by the authors in Reference [30] for cruciform welded joints. Particular values of the exponents, *m* and *p*, and coefficients *B*_1_-*B*_4_, were determined for each loading mode using the least squares method. The best fitting of the functions *κ*_t_, *κ*_b_ and *κ*_s_, to the numerical FEM SCF results were obtained for *m* = 1, for tensile and bending load, and for *m* = 2 for shearing load, and for *p* equal to 2.4, 2.6, and 2.0 for tensile, bending, and shearing loads, respectively. Unfortunately, the coefficients *B*_i_ depended also on the weld angle *θ*. Therefore, additional approximations for *B*_i_ = f_i_(*θ*) were necessary. Mathematical representations of the correction functions *κ*_t_, *κ*_b_, and *κ*_s_, suitable for each loading mode, are given in Appendix A. Some examples of the correction functions *κ*_t_, *κ*_b_, and *κ*_s_ for tensile, bending, and shearing loads, respectively, are shown in Figure 10, Figure 11 and Figure 12, for arbitrarily chosen parameters: *θ* = 45° and *T*/*a* equal to 2 and 4.

### 4.4. Validation of SCF Approximation Functions

Accuracy of the SCF approximation functions, given by the Formulas (A1)–(A3), containing correction functions *κ*_t_, *κ*_b_, and *κ*_s_, > 1, was verified for all loading modes and for the weld angle *θ* varying in the range of 30–60°. Some examples of the validation are presented in Table 5, Table 6 and Table 7 for arbitrarily chosen angles *θ* equal to 30°, 45°, and 55°.

Similar validation was made for other weld angles *θ* with a step of 2.5°. The maximum percentage error found for all analysed cases was lower than 2% with respect to the SCFs’ results obtained numerically using the FEM. Due to the fact that the weld angle *θ* = 45° is commonly used in engineering applications of such T-joints, extended parametric Equations (A1)–(A3) given in Appendix A, were reduced to Equations (A4)–(A6) presented in Appendix B.

## 5. Discussion

**Figure 13:**
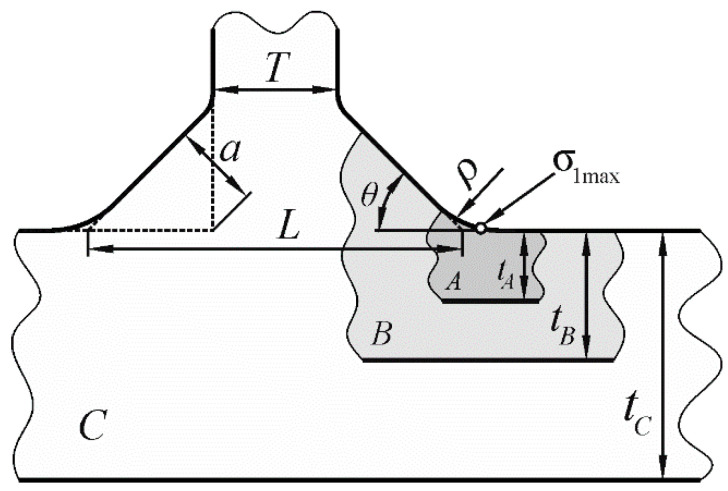
Characteristic dimensional proportions representing the influence of characteristic geometrical dimensions on SCF.

Several thousand SCF solutions obtained using the FEM as well as corresponding parametric Equations (A1)–(A3) of high accuracy and a wide range of validity, made it possible to draw some more general conclusions regarding the influence of particular geometrical parameters on SCF. The general rule is that particular characteristic geometrical details of the joint located closer to the reference point of the maximum stress σ_1max_ affect this value more significantly than others. In order to explain this phenomenon more precisely, three qualitatively different geometrical cases of a T-joint are shown in Figure 13. Additionally, some alternative, parametric SCF formulas available in the literature [13,15,16,17,19] and related to T-joints are also given in Appendix C.

In case A, shown in Figure 13, the main plate is relatively thin compared to other dimensions of the joint, *t*_A_ << *L*, while the magnitude of the weld toe radius *ρ* is of the same order as the plate thickness *t*_A_. In such a case, SCF values depend mainly on the *t*_A_/*ρ* ratio. For example, when *t*_A_/*ρ* changes in the range of 0.8–5.0, *K_tt_* varies from 1.16 to 1.63 and does not depend on the weld angle *θ* in the range of 30–60°. Similar behaviour is observed for *t*_A_/*ρ* in the range of 0.8–4.0, where *K_tb_* varies from 1.16 to 1.66. In case B, when the weld toe radius *ρ* is much smaller than in the previous case and *L* >> *t*_B_, SCF values should depend on the quantities *t*_B_/*ρ* and *θ*.

Equations (A9) and (A12) presented in Appendix C and numerical SCF data shown in Table 8 confirm such a conclusion.

However, accuracy of the formulas given in Appendix C depends on the mathematical representation chosen by the authors, assumed range of validity, and the accuracy of data used in approximations. Some comparisons of SCFs obtained from various equations to the FEM results carried out in the present work are shown in Table 9 and Table 10 for *θ* = 45°.

In case C, shown in Figure 13, mutual relations between *L* and *t*_C_ become important, especially when the *L* is much shorter than *t*_C_. The presence of such a quantity, *L*/*t*_C_, can be observed directly in Equations (A10) and (A14). In a general case, such an influencing parameter depends on the thickness of the stiffener, *T*, leg lengths, weld angle *θ*, and the weld toe radius *ρ*. Therefore, it seems reasonable to use all these quantities in order to derive “effective length” of the joint and, as a consequence, reach better SCF approximations than by a simple use of *L*. It is also worth noting that SCFs used in Equations (A10) and (A14) are defined in a different manner than in other formulas presented in this work. They are related to the maximum longitudinal stress component at the weld toe and not to the maximum principal stress σ_1max_. Therefore, SCF values obtained from Equations (A10) and (A14) and shown in Table 9 and Table 10, are a few percent underestimated compared with appropriate FEM results.

Particular data given in Table 9 and Table 10 represent only some comparative examples of SCF values with respect to the FEM results. Therefore, these results cannot be generalised to assess accuracy of each formula. Some of these results denoted by (#) mean that they formally lie out of range of validity assumed by the authors. However, in many cases, the accuracy remains satisfactory. 

It may be assumed that the range of validity of the formulas (A8) and (A13), proposed by Tsuji [17], is similar to that for Equations (A7) and (A11) reported by Ushirokawa and Nakayama [16].

## 6. Conclusions

Systematic numerical FEM modelling including more than 22,600 cases of welded plate T-joints made it possible to derive three approximating SCF’s formulas for axial, bending, and shearing loads. Five geometrical parameters: *ρ*, *a*, *θ*, *t*, and *T* were considered as independent variables varying in relatively wide ranges 0 < *ρ*/*a* ≤ 1.3, 0 < *a*/*t* ≤ 1.3, 1 ≤ *T*/*a* ≤ 4, and 30° ≤ *θ* ≤ 60° covering dimensional proportions of welded plate T-joints used in engineering applications. Two limiting cases are also included in the solutions—when the weld toe radius tends to zero and when the main plate thickness becomes infinite. The accuracy of the formulas is better than 98% compared with numerical FEM results. The use of the proper exponents, *n* and *n*_s_, corresponding to the stress field singularities of a sharp corner dependent on the angle *θ*, was the key condition in obtaining such an accuracy and range of validity of proposed parametric equations. In the cases of shearing loads, a plane FEM model based on thermal analogy was successfully used.

In spite of the fact that SCF alone is not sufficient for calculating fatigue strength of a structural element, all the parametric formulas given in a closed form may be used as a part of computer-aided procedures for the fatigue strength assessment of welded plate T-joints.

Presented solutions may be applied as a tool for a computer aided assessment in fatigue design of welded plate T-joints. Particularly in:

(a) comparative studies of stress concentration in various geometrical forms of welded T-joints, including the use of fictitious values of the weld toe radii,
(b) hot spot method applied in fatigue design of such joints,
(c) weight function method used in fracture mechanics models for crack initiated at the weld toe,
(d) dealing with the necessity of additional mechanical improvements in the weld toe region.

## Figures and Tables

**Table 1:** Representation of SCF values for a plate T-joint subjected to shearing load, while *θ* = 45° and *T*/*a* = 1.

*θ* = 45°	*X*															
*Y*	0.010	0.018	0.025	0.032	0.050	0.079	0.100	0.150	0.200	0.250	0.300	0.350	0.400	0.450	0.500	0.562
0.010	3.474	3.093	2.886	2.750	2.500	2.266	2.154	1.966	1.837	1.738	1.657	1.588	1.528	1.475	1.427	1.372
0.018	3.472	3.092	2.885	2.749	2.500	2.265	2.154	1.966	1.837	1.738	1.657	1.588	1.528	1.475	1.427	1.372
0.025	3.470	3.091	2.884	2.749	2.499	2.265	2.154	1.966	1.836	1.737	1.656	1.588	1.528	1.475	1.426	1.372
0.032	3.469	3.090	2.884	2.748	2.499	2.265	2.154	1.966	1.836	1.737	1.656	1.588	1.528	1.474	1.426	1.371
0.050	3.464	3.088	2.881	2.746	2.497	2.263	2.152	1.964	1.835	1.736	1.655	1.586	1.527	1.473	1.425	1.370
0.079	3.455	3.079	2.876	2.740	2.492	2.258	2.148	1.960	1.830	1.732	1.652	1.583	1.523	1.470	1.422	1.367
0.100	3.453	3.076	2.871	2.735	2.487	2.254	2.143	1.956	1.827	1.729	1.648	1.580	1.520	1.467	1.419	1.364
0.150	3.429	3.053	2.849	2.715	2.468	2.237	2.127	1.941	1.813	1.715	1.636	1.568	1.508	1.456	1.408	1.353
0.200	3.389	3.018	2.816	2.683	2.440	2.211	2.102	1.919	1.792	1.695	1.616	1.549	1.491	1.439	1.391	1.337
0.250	3.335	2.970	2.771	2.641	2.401	2.176	2.069	1.888	1.764	1.669	1.591	1.525	1.467	1.416	1.369	1.316
0.300	3.269	2.911	2.715	2.588	2.353	2.132	2.028	1.850	1.729	1.635	1.559	1.495	1.438	1.388	1.343	1.291
0.350	3.191	2.841	2.650	2.526	2.296	2.081	1.979	1.806	1.688	1.597	1.523	1.460	1.405	1.357	1.313	1.263
0.400	3.104	2.763	2.578	2.457	2.234	2.024	1.925	1.757	1.642	1.554	1.483	1.422	1.369	1.323	1.281	1.233
0.450	3.009	2.679	2.500	2.383	2.166	1.963	1.867	1.705	1.594	1.509	1.440	1.382	1.332	1.288	1.248	1.204
0.500	2.911	2.592	2.418	2.304	2.095	1.899	1.807	1.650	1.543	1.462	1.396	1.341	1.294	1.252	1.216	1.175
0.562	2.782	2.477	2.312	2.203	2.003	1.816	1.728	1.579	1.478	1.402	1.341	1.290	1.247	1.210	1.177	1.142

**Table 2:** Comparison of *K_tt_* values calculated using Equation (A1) (*) to the corresponding FEM results for various *X* and *Y*, while *θ* = 45° and *T*/*a* = 1.

*θ* = 45°	*X*						
*Y*	0.050	0.100	0.200	0.300	0.400	0.500	0.562
0.050	3.916 3.930 *	3.091 3.099 *	2.412 2.419 *	2.070 2.075 *	1.845 1.850 *	1.679 1.683 *	1.593 1.596 *
0.100	3.848 3.854 *	3.034 3.039 *	2.369 2.372 *	2.033 2.035 *	1.812 1.813 *	1.648 1.647 *	1.562 1.561 *
0.200	3.540 3.530 *	2.793 2.785 *	2.183 2.177 *	1.875 1.870 *	1.672 1.668 *	1.520 1.517 *	1.440 1.439 *
0.300	3.099 3.108 *	2.451 2.459 *	1.927 1.933 *	1.666 1.671 *	1.496 1.500 *	1.371 1.374 *	1.307 1.309 *
0.400	2.696 2.702 *	2.145 2.150 *	1.708 1.711 *	1.496 1.497 *	1.361 1.361 *	1.263 1.262 *	1.214 1.212 *
0.500	2.379 2.374 *	1.910 1.906 *	1.545 1.542 *	1.373 1.371 *	1.265 1.264 *	1.189 1.190 *	1.152 1.153 *
0.562	2.209 2.216 *	1.784 1.790 *	1.460 1.464 *	1.309 1.311 *	1.216 1.218 *	1.152 1.152 *	1.121 1.121 *

**Table 3:** Comparison of *K_tb_* values calculated using Equation (A2) (*) to the corresponding FEM results for various *X* and *Y*, while *T*/*a* = 1 and *θ* = 45°.

*θ* = 45°	*X*						
*Y*	0.050	0.100	0.200	0.300	0.400	0.500	0.562
0.050	3.997 4.010 *	3.149 3.160 *	2.455 2.463 *	2.103 2.110 *	1.872 1.878 *	1.700 1.706 *	1.611 1.616 *
0.100	4.020 4.027 *	3.164 3.171 *	2.462 2.467 *	2.104 2.108 *	1.868 1.872 *	1.692 1.695 *	1.601 1.602 *
0.200	3.868 3.856 *	3.041 3.033 *	2.358 2.351 *	2.007 2.001 *	1.773 1.768 *	1.596 1.593 *	1.505 1.501 *
0.300	3.494 3.502 *	2.746 2.753 *	2.127 2.131 *	1.806 1.813 *	1.595 1.602 *	1.440 1.444 *	1.357 1.363 *
0.400	3.059 3.072 *	2.411 2.416 *	1.873 1.876 *	1.599 1.604 *	1.423 1.428 *	1.298 1.302 *	1.236 1.240 *
0.500	2.685 2.671 *	2.118 2.106 *	1.659 1.649 *	1.435 1.426 *	1.297 1.290 *	1.204 1.198 *	1.161 1.157 *
0.562	2.469 2.482 *	1.952 1.963 *	1.540 1.548 *	1.346 1.352 *	1.231 1.236 *	1.157 1.161 *	1.123 1.126 *

**Table 4:** Comparison of *K_ts_* values calculated using Equation (A3) (*) to the corresponding FEM results for various *X* and *Y*, while *T*/*a* = 1 and *θ* = 45°.

*θ* = 45°	*X*						
*Y*	0.050	0.100	0.200	0.300	0.400	0.500	0.562
0.050	2.497 2.486 *	2.152 2.143 *	1.835 1.827 *	1.655 1.648 *	1.527 1.520 *	1.425 1.418 *	1.370 1.363 *
0.100	2.487 2.474 *	2.143 2.133 *	1.827 1.818 *	1.648 1.640 *	1.520 1.513 *	1.419 1.412 *	1.364 1.357 *
0.200	2.440 2.426 *	2.102 2.092 *	1.792 1.784 *	1.616 1.609 *	1.491 1.485 *	1.391 1.387 *	1.337 1.334 *
0.300	2.353 2.346 *	2.028 2.023 *	1.729 1.726 *	1.559 1.558 *	1.438 1.439 *	1.343 1.346 *	1.291 1.295 *
0.400	2.234 2.235 *	1.925 1.927 *	1.642 1.645 *	1.483 1.486 *	1.369 1.375 *	1.281 1.288 *	1.233 1.241 *
0.500	2.095 2.091 *	1.807 1.804 *	1.543 1.541 *	1.396 1.394 *	1.294 1.291 *	1.216 1.213 *	1.175 1.172 *
0.562	2.003 1.986 *	1.728 1.713 *	1.478 1.464 *	1.341 1.326 *	1.247 1.230 *	1.177 1.158 *	1.142 1.121 *

**Table 5:** Comparison of SCF’s values calculated using Equations (A1)–(A3) (*) to the corresponding FEM results for various *T*/*a* ratios, while *θ* = 30°.

*θ* = 30°		*K_tt_*				*K_tb_*				*K*_*ts*_			
*ρ*/*a*	*t*/*a*	*T*/*a* = 1	*T*/*a* = 2	*T*/*a* = 3	*T*/*a* = 4	*T*/*a* = 1	*T*/*a* = 2	*T*/*a* = 3	*T*/*a* = 4	*T*/*a* = 1	*T*/*a* = 2	*T*/*a* = 3	*T*/*a* = 4
0.05	10	3.469 3.489 *	3.588 3.613 *	3.681 3.708 *	3.754 3.788 *	3.556 3.571 *	3.698 3.721 *	3.813 3.836 *	3.907 3.933 *	2.144 2.156 *	2.203 2.224 *	2.258 2.277 *	2.306 2.321 *
	7	3.378 3.394 *	3.462 3.471 *	3.519 3.529 *	3.557 3.578 *	3.499 3.506 *	3.607 3.612 *	3.687 3.694 *	3.746 3.763 *	2.133 2.146 *	2.186 2.207 *	2.234 2.253 *	2.276 2.292 *
	4	3.110 3.129 *	3.129 3.141 *	3.136 3.151 *	3.138 3.158 *	3.278 3.284 *	3.314 3.309 *	3.331 3.328 *	3.337 3.345 *	2.092 2.113 *	2.130 2.154 *	2.162 2.185 *	2.186 2.212 *
0.25	10	2.334 2.347 *	2.413 2.426 *	2.474 2.487 *	2.522 2.538 *	2.391 2.400 *	2.485 2.498 *	2.561 2.574 *	2.624 2.638 *	1.705 1.714 *	1.752 1.768 *	1.793 1.809 *	1.833 1.844 *
	7	2.273 2.283 *	2.328 2.331 *	2.366 2.368 *	2.390 2.399 *	2.352 2.356 *	2.424 2.425 *	2.477 2.478 *	2.515 2.523 *	1.696 1.706 *	1.738 1.754 *	1.775 1.791 *	1.808 1.822 *
	4	2.094 2.107 *	2.106 2.114 *	2.110 2.120 *	2.111 2.124 *	2.204 2.208 *	2.227 2.224 *	2.238 2.236 *	2.242 2.246 *	1.663 1.680 *	1.694 1.712 *	1.718 1.737 *	1.738 1.758 *
0.5	10	1.976 1.987 *	2.040 2.051 *	2.090 2.101 *	2.129 2.142 *	2.022 2.028 *	2.098 2.109 *	2.161 2.172 *	2.214 2.225 *	1.546 1.552 *	1.588 1.600 *	1.626 1.637 *	1.661 1.668 *
	7	1.925 1.934 *	1.969 1.972 *	1.999 2.001 *	2.019 2.026 *	1.988 1.991 *	2.047 2.047 *	2.090 2.091 *	2.122 2.127 *	1.538 1.545 *	1.575 1.588 *	1.609 1.621 *	1.639 1.648 *
	4	1.775 1.788 *	1.784 1.793 *	1.787 1.797 *	1.788 1.801 *	1.863 1.866 *	1.881 1.878 *	1.890 1.888 *	1.892 1.896 *	1.508 1.522 *	1.535 1.551 *	1.557 1.572 *	1.575 1.591 *
1	10	1.687 1.696 *	1.737 1.748 *	1.776 1.788 *	1.807 1.821 *	1.723 1.728 *	1.783 1.796 *	1.832 1.848 *	1.874 1.892 *	1.405 1.409 *	1.442 1.452 *	1.475 1.484 *	1.506 1.512 *
	7	1.643 1.652 *	1.677 1.682 *	1.700 1.705 *	1.715 1.725 *	1.693 1.695 *	1.738 1.741 *	1.772 1.777 *	1.796 1.807 *	1.398 1.403 *	1.430 1.441 *	1.460 1.469 *	1.486 1.494 *
	4	1.519 1.534 *	1.525 1.537 *	1.527 1.540 *	1.527 1.543 *	1.584 1.588 *	1.597 1.598 *	1.603 1.605 *	1.604 1.611 *	1.371 1.383 *	1.394 1.408 *	1.413 1.427 *	1.429 1.443 *

**Table 6:** Comparison of SCF’s values calculated using Equations (A1)–(A3) (*) to the corresponding FEM results for various *T*/*a* ratios, while *θ* = 45°.

***θ*** **= 45°**		***K*** **_*tt*_**				***K*** **_*tb*_**				***K*** **_*ts*_**			
***ρ*** **/*a***	***t*** **/*a***	***T*** **/*a* = 1**	***T*** **/*a* = 2**	***T*** **/*a* = 3**	***T*** **/*a* = 4**	***T*** **/*a* = 1**	***T*** **/*a* = 2**	***T*** **/*a* = 3**	***T*** **/*a* = 4**	***T*** **/*a* = 1**	***T*** **/*a* = 2**	***T*** **/*a* = 3**	***T*** **/*a* = 4**
0.05	10	3.940 3.938 *	4.200 4.200 *	4.404 4.402 *	4.565 4.572 *	4.095 4.097 *	4.409 4.429 *	4.670 4.684 *	4.889 4.898 *	2.514 2.502 *	2.632 2.630 *	2.735 2.728 *	2.824 2.811 *
	7	3.865 3.853 *	4.078 4.048 *	4.229 4.197 *	4.331 4.323 *	4.081 4.074 *	4.364 4.353 *	4.582 4.568 *	4.749 4.748 *	2.504 2.490 *	2.615 2.608 *	2.709 2.698 *	2.789 2.774 *
	4	3.609 3.588 *	3.701 3.650 *	3.740 3.697 *	3.754 3.736 *	3.941 3.921 *	4.107 4.053 *	4.203 4.155 *	4.256 4.240 *	2.464 2.451 *	2.554 2.540 *	2.623 2.608 *	2.677 2.665 *
0.25	10	2.380 2.384 *	2.523 2.525 *	2.636 2.634 *	2.727 2.725 *	2.456 2.470 *	2.630 2.651 *	2.776 2.790 *	2.899 2.907 *	1.829 1.820 *	1.913 1.911 *	1.986 1.981 *	2.049 2.040 *
	7	2.335 2.333 *	2.451 2.436 *	2.533 2.514 *	2.589 2.581 *	2.450 2.451 *	2.606 2.601 *	2.727 2.715 *	2.820 2.812 *	1.821 1.812 *	1.900 1.895 *	1.967 1.959 *	2.024 2.013 *
	4	2.183 2.177 *	2.230 2.207 *	2.250 2.230*	2.257 2.249 *	2.358 2.351 *	2.411 2.447 *	2.492 2.468 *	2.520 2.511 *	1.792 1.784 *	1.855 1.846 *	1.905 1.894 *	1.943 1.935 *
0.5	10	1.959 1.962 *	2.063 2.066 *	2.147 2.145 *	2.215 2.212 *	2.012 2.024 *	2.140 2.158 *	2.248 2.262 *	2.340 2.349 *	1.603 1.596 *	1.673 1.672 *	1.734 1.730 *	1.789 1.780 *
	7	1.922 1.920 *	2.005 1.993 *	2.063 2.050 *	2.104 2.097 *	1.999 2.004 *	2.110 2.113 *	2.199 2.196 *	2.266 2.267 *	1.596 1.588 *	1.662 1.658 *	1.718 1.711 *	1.766 1.756 *
	4	1.799 1.795 *	1.830 1.814 *	1.842 1.829 *	1.847 1.842 *	1.915 1.915 *	1.976 1.959 *	2.010 1.993 *	2.029 2.022 *	1.571 1.564 *	1.623 1.616 *	1.664 1.655 *	1.696 1.689 *
1	10	1.655 1.656 *	1.726 1.730 *	1.783 1.787 *	1.829 1.835 *	1.690 1.699 *	1.777 1.798 *	1.852 1.874 *	1.916 1.937 *	1.420 1.414 *	1.475 1.475 *	1.524 1.522 *	1.569 1.562 *
	7	1.623 1.620 *	1.676 1.670 *	1.715 1.709 *	1.741 1.741 *	1.673 1.677 *	1.747 1.754 *	1.805 1.813 *	1.851 1.863 *	1.414 1.408 *	1.465 1.463 *	1.510 1.506 *	1.549 1.542 *
	4	1.520 1.517 *	1.536 1.528 *	1.543 1.536 *	1.544 1.543 *	1.596 1.593 *	1.627 1.621 *	1.646 1.642 *	1.655 1.659 *	1.391 1.387 *	1.431 1.428 *	1.462 1.458 *	1.488 1.484 *

**Table 7:** Comparison of SCF’s values calculated using Equations (A1)–(A3) (*) to the corresponding FEM results for various *T/a* ratios, while *θ* = 55°.

*θ* = 55°		*K*_*tt*_				*K*_*tb*_				*K*_*ts*_			
*ρ*/*a*	*t*/*a*	*T*/*a* = 1	*T*/*a* = 2	*T*/*a* = 3	*T*/*a* = 4	*T*/*a* = 1	*T*/*a* = 2	*T*/*a* = 3	*T*/*a* = 4	*T*/*a* = 1	*T*/*a* = 2	*T*/*a* = 3	*T*/*a* = 4
0.05	10	4.005 4.026 *	4.339 4.367 *	4.604 4.628 *	4.816 4.848 *	4.180 4.198 *	4.591 4.641 *	4.939 4.981 *	5.234 5.267 *	2.714 2.716 *	2.875 2.888 *	3.011 3.020 *	3.127 3.132 *
	7	3.941 3.951 *	4.225 4.216 *	4.430 4.419 *	4.574 4.590 *	4.193 4.220 *	4.579 4.593 *	4.887 4.894 *	5.129 5.147 *	2.703 2.703 *	2.857 2.862 *	2.983 2.985 *	3.089 3.089 *
	4	3.707 3.703 *	3.849 3.802 *	3.915 3.879 *	3.938 3.943 *	4.115 4.110 *	4.383 4.336 *	4.549 4.509 *	4.649 4.656 *	2.663 2.659 *	2.791 2.784 *	2.890 2.880 *	2.965 2.961 *
0.25	10	2.347 2.359 *	2.512 2.529 *	2.646 2.659 *	2.753 2.769 *	2.426 2.437 *	2.631 2.658 *	2.807 2.828 *	2.958 2.972 *	1.878 1.879 *	1.984 1.993 *	2.074 2.082 *	2.154 2.156 *
	7	2.309 2.315 *	2.448 2.444 *	2.548 2.543 *	2.618 2.627 *	2.423 2.429 *	2.614 2.621 *	2.767 2.767 *	2.889 2.891 *	1.870 1.870 *	1.971 1.976 *	2.055 2.058 *	2.126 2.127 *
	4	2.175 2.175 *	2.239 2.219 *	2.268 2.254 *	2.278 2.283 *	2.358 2.356 *	2.484 2.459 *	2.562 2.537 *	2.608 2.603 *	1.842 1.840 *	1.926 1.923 *	1.991 1.986 *	2.040 2.040 *
0.5	10	1.931 1.940 *	2.046 2.059 *	2.139 2.149 *	2.215 2.226 *	1.984 1.991 *	2.126 2.146 *	2.250 2.265 *	2.356 2.366 *	1.620 1.620 *	1.705 1.712 *	1.778 1.782 *	1.842 1.842 *
	7	1.899 1.904 *	1.993 1.991 *	2.061 2.058 *	2.108 2.114 *	1.974 1.978 *	2.104 2.108 *	2.208 2.208 *	2.291 2.293 *	1.613 1.612 *	1.694 1.697 *	1.761 1.762 *	1.819 1.817 *
	4	1.789 1.790 *	1.828 1.817 *	1.845 1.838 *	1.851 1.855 *	1.907 1.904 *	1.986 1.968 *	2.034 2.016 *	2.062 2.057 *	1.589 1.588 *	1.655 1.653 *	1.706 1.702 *	1.745 1.745 *
1	10	1.642 1.648 *	1.716 1.726 *	1.777 1.786 *	1.826 1.836 *	1.676 1.683 *	1.768 1.787 *	1.848 1.868 *	1.917 1.935 *	1.424 1.422 *	1.486 1.491 *	1.541 1.544 *	1.590 1.589 *
	7	1.612 1.616 *	1.670 1.670 *	1.711 1.711 *	1.740 1.746 *	1.661 1.664 *	1.740 1.747 *	1.805 1.811 *	1.856 1.865 *	1.418 1.416 *	1.476 1.479 *	1.526 1.527 *	1.570 1.568 *
	4	1.516 1.520 *	1.534 1.532 *	1.542 1.542 *	1.545 1.550 *	1.587 1.586 *	1.627 1.618 *	1.651 1.643 *	1.663 1.664 *	1.396 1.396 *	1.441 1.442 *	1.478 1.478 *	1.507 1.508 *

**Table 8:** Influence of the weld angle *θ* and *t*/*ρ* ratio on SCF for *L* > 4*t*.

	***θ***	***t*** **/*ρ***												
		**0.8**	**1.0**	**1.5**	**2.0**	**3.0**	**4.0**	**5.0**	**10.0**	**20.0**	**35.0**	**50.0**	**75.0**	**100.0**
*K*_*tt*_	30°	1.16	1.19	1.27	1.33	1.44	1.53	1.61	1.89	2.24	2.57	2.81	3.10	3.33
	45°	1.16	1.19	1.26	1.33	1.44	1.54	1.63	1.96	2.41	2.87	3.21	3.65	4.01
	60°	1.16	1.19	1.26	1.33	1.44	1.54	1.63	1.96	2.44	2.93	3.32	3.85	4.28
*K*_*tb*_	30°	1.16	1.20	1.30	1.37	1.50	1.60	1.68	1.99	2.35	2.70	2.95	3.27	3.51
	45°	1.16	1.20	1.29	1.37	1.52	1.65	1.76	2.18	2.72	3.25	3.65	4.17	4.58
	60°	1.16	1.20	1.29	1.37	1.53	1.66	1.79	2.27	2.93	3.61	4.13	4.83	5.39

**Table 9:** Accuracy of Equations (A1) and (A7)–(A10) for a T-joint subjected to tension, where: *θ* = 45°, *h* = *h*_p_, *t* = *T*, *h*/*t* = 0.75, 0.025 ≤ *ρ*/*t* ≤ 0.35, #—out of range.

	*ρ*/*t*	0.025	0.050	0.075	0.10	0.15	0.25	0.35
*K_tt_*	FEM	3.002	2.417	2.139	1.967	1.757	1.544	1.430
	U and N (A7)	2.885	2.202	1.923	1.766	1.588	1.422	1.339
	Tsuji (A8)	2.426	2.047	1.874	1.769	1.641	1.511	1.440
	Monahan (A9)	2.894	2.383	# 2.150	# 2.009	# 1.840	# 1.666	# 1.571
	Brennan et al. (A10)	2.749	2.270	# 2.055	# 1.925	# 1.769	# 1.611	# 1.525
	(A1)	3.015	2.428	2.148	1.975	1.764	1.549	1.433

**Table 10:** Accuracy of Equations (A2) and (A11)–(A14) for the T-joint subjected to bending, where: *θ* = 45°, *h* = *h*_p_, *t* = *T*, *h*/*t* = 0.75, 0.025 ≤ *ρ*/*t* ≤ 0.35, #—out of range.

	*ρ*/*t*	0.025	0.050	0.075	0.10	0.15	0.25	0.35
*K_tb_*	FEM	3.434	2.740	2.404	2.195	1.935	1.663	1.516
	U and N (A11)	3.327	2.750	2.444	2.238	1.957	1.625	1.436
	Niu and Glinka (A12)	# 3.516	# 2.818	# 2.503	# 2.313	# 2.086	# 1.855	# 1.730
	Tsuji (A13)	3.471	2.833	2.539	2.359	2.142	1.916	1.792
	Brennan et al. (A14)	3.217	2.606	# 2.330	# 2.164	# 1.964	# 1.761	# 1.652
	(A2)	3.430	2.741	2.408	2.199	1.940	1.669	1.523

## Data Availability

Data is contained within the article.

## Nomenclature

*a*	theoretical weld throat thickness
*G*	shear modulus
*h*, *h*_p_	leg lengths defining the weld size
*k*	thermal conductivity
*K*_t_	stress concentration factor (SCF)
*K_tt_*	stress concentration factor for tensile (axial) load
*K*_*tb*_	stress concentration factor for bending load
*K*_*ts*_	stress concentration factor for shearing load
*m*, *p*	exponents dependent on the loading mode
*n*	stress field exponent for a sharp corner for axial and bending load
*n*_s_	stress field exponent for a sharp corner subjected to shearing load
*P*	regular function represented by polynomials
*P*_t_, *P*_b_, *P*_s_	functions *P* corresponding to tensile, bending, and shearing load, respectively
*q*	magnitude of the heat flux
*q*_max_	magnitude of the maximum heat flux
*q*_nom_	magnitude of the nominal heat flux at the end of the body
*t*	thickness of the main plate
*T*	thickness of the attachment plate
*T*_temp_	temperature
*W*	displacement component corresponding to the anti–plane shear
*X* = *ρ*/(*ρ* + *a*)	normalized toe radius parameter
*Y* = *a*/(*a* + *t*)	normalized weld thickness parameter
*Z* = *T*/*a*	normalized attachment plate thickness parameter
*Z*_0_	arbitrarily chosen reference *T*/*a* value
|∇*Ψ*|	magnitude of the displacement (temperature) gradient
|∇*Ψ*|_max_	magnitude of the maximum displacement (temperature) gradient
|∇*Ψ*|_nom_	magnitude of the nominal displacement (temperature) gradient
*θ*	weld angle
*κ*	correction function for the relative attachment plate thickness *T*/*a*
*κ*_t_	correction function κ for tensile (axial) load
*κ*_b_	correction function κ for bending load
*κ*_s_	correction function κ for shearing load
*Ψ*(*x,y*)	potential function representing temperature or anti–plane displacement
*∂Ψ*/*∂*n	normal derivative at the bounding contour
*ρ*	weld toe radius
*σ*_1_	first principal stress produced by normal or bending load
*σ*_1max_	maximum value of the first principal stress at the weld toe zone
*σ*_t_	nominal tensile (axial) stress
*σ*_b_	nominal bending stress
*τ*_max_	maximum shear stress at the weld toe due to shearing load
*τ*_s_	nominal shear stress

## Appendix A

### Approximating SCFs’ Formulas for a Welded Plate T-Joint Subjected to Axial, Bending, and Shearing Loads

Independent variables: *ρ*, *a*, *θ*, *t*, *T*;

Normalized quantities: *X* = *ρ*/(*ρ* + *a*), *Y* = *a*/(*a* + *t*); *Z* = *T*/*a*;

Range of validity: 0 < *ρ*/*a* ≤ 1.3; 0 < *a*/*t* ≤ 1.3; 1 ≤ *T*/*a* ≤ 4; 30° ≤ *θ* ≤ 60°.

In all formulas, *θ* is expressed in radians.

Exponent of the singular term for:

Axial and bending load:

$$n=\frac{-0.63662\theta -0.09330\theta^{2}}{1+0.77635\theta +0.04075\theta^{1.5}-0.00499\theta^{2}+0.13365\theta^{2.5}}$$

Shearing load:

$$n_{s}=\frac{-\theta }{\theta +\pi }$$

Accuracy: maximum percentage error lower than 2% compared to the FEM results

Tensile (axial) load:

(A1)
$$K_{tt}=X^{n}\left(A_{0}^{t}+A_{1}^{t}X+A_{2}^{t}X^{2}+A_{3}^{t}X^{3}+A_{4}^{t}X^{4}\right)\kappa_{t}$$

where:

$$A_{0}^{t}=A_{00}^{t}+A_{01}^{t}Y+A_{02}^{t}Y^{2}+A_{03}^{t}Y^{3}+A_{04}^{t}Y^{4}$$

$$A_{00}^{t}=2.078-0.712\theta -0.076\theta^{4}$$

$$A_{01}^{t}=0.132+0.718\theta -0.455\theta^{4}$$

$$A_{02}^{t}=-18.982+12.585\theta +0.398\theta^{4}$$

$$A_{03}^{t}=55.711-54.642\theta +5.304\theta^{4}$$

$$A_{04}^{t}=-47.047+53.604\theta -7.139\theta^{4}$$

$$A_{1}^{t}=A_{10}^{t}+A_{11}^{t}Y+A_{12}^{t}Y^{2}+A_{13}^{t}Y^{3}+A_{14}^{t}Y^{4}$$

$$A_{10}^{t}=-0.066-0.789\theta +0.878\theta^{4}$$

$$A_{11}^{t}=-0.413+0.119\theta^{2}+0.428\theta^{4}$$

$$A_{12}^{t}=6.193-5.495\theta^{2}-5.077\theta^{4}$$

$$A_{13}^{t}=-20.187+34.745\theta^{2}+11.092\theta^{4}$$

$$A_{14}^{t}=16.393-27.986\theta^{2}-13.135\theta^{4}$$

$$A_{2}^{t}=A_{20}^{t}+A_{21}^{t}Y+A_{22}^{t}Y^{2}+A_{23}^{t}Y^{3}+A_{24}^{t}Y^{4}$$

$$A_{20}^{t}=5.133-21.927\theta +24.944\theta^{2}-8.229\theta^{4}$$

$$A_{21}^{t}=2.250-2.429\theta^{2}+0.805\theta^{4}$$

$$A_{22}^{t}=-5.156-6.961\theta^{2}+14.020\theta^{4}$$

$$A_{23}^{t}=0.909+92.878\theta^{2}-118.392\theta^{4}$$

$$A_{24}^{t}=16.571-147.711\theta^{2}+151.148\theta^{4}$$

$$A_{3}^{t}=A_{30}^{t}+A_{31}^{t}Y+A_{32}^{t}Y^{2}+A_{33}^{t}Y^{3}+A_{34}^{t}Y^{4}$$

$$A_{30}^{t}=-15.018+58.059\theta -60.616\theta^{2}+17.595\theta^{4}$$

$$A_{31}^{t}=-7.053+5.113\theta -0.340\theta^{4}$$

$$A_{32}^{t}=14.167+8.281\theta^{2}-22.438\theta^{4}$$

$$A_{33}^{t}=19.091-213.131\theta^{2}+226.174\theta^{4}$$

$$A_{34}^{t}=-146.976+316.815\theta -195.919\theta^{4}$$

$$A_{4}^{t}=A_{40}^{t}+A_{41}^{t}Y+A_{42}^{t}Y^{2}+A_{43}^{t}Y^{3}+A_{44}^{t}Y^{4}$$

$$A_{40}^{t}=10.494-40.594\theta +41.995\theta^{2}-11.917\theta^{4}$$

$$A_{41}^{t}=24.260-73.105\theta +67.325\theta^{2}-17.427\theta^{4}$$

$$A_{42}^{t}=-1.928-16.706\theta^{2}+18.955\theta^{4}$$

$$A_{43}^{t}=-86.411+181.383\theta -108.284\theta^{4}$$

$$A_{44}^{t}=117.729-227.646\theta +117.488\theta^{4}$$

$$\begin{array}{l} \kappa_{t}=1+\left(\sqrt{Z}-1\right)\left\{1-\left[\left(-0.889+2.279\theta -0.539\theta^{2}\right)+\left(12.70+10.21\theta -7.17\theta^{2}\right)Y^{2}\right]X\right\}\cdot \\ \;\;\cdot Exp\left\{-{\left[\left(12.94-13.94\theta +6.57\theta^{2}\right)Y\right]}^{2.4}-\left(3.72-4.03\theta +1.62\theta^{2}\right)\right\} \end{array}$$

Bending load:

(A2)
$$K_{tb}=X^{n}\left(A_{0}^{b}+A_{1}^{b}X+A_{2}^{b}X^{2}+A_{3}^{b}X^{3}+A_{4}^{b}X^{4}\right)\kappa_{b}$$

where:

$$A_{0}^{b}=A_{00}^{b}+A_{01}^{b}Y+A_{02}^{b}Y^{2}+A_{03}^{b}Y^{3}+A_{04}^{b}Y^{4}$$

$$A_{00}^{b}=1.833-0.316\theta^{2}-0.621\theta^{3}+0.394\theta^{4}$$

$$A_{01}^{b}=-1.282+6.636\theta -10.422\theta^{3}+5.974\theta^{4}$$

$$A_{02}^{b}=-16.721-7.442\theta^{2}+54.668\theta^{3}-33.383\theta^{4}$$

$$A_{03}^{b}=50.505-118.407\theta^{2}+50.936\theta^{3}+12.039\theta^{4}$$

$$A_{04}^{b}=-43.771+162.845\theta^{2}-140.901\theta^{3}+30.243\theta^{4}$$

$$A_{1}^{b}=A_{10}^{b}+A_{11}^{b}Y+A_{12}^{b}Y^{2}+A_{13}^{b}Y^{3}+A_{14}^{b}Y^{4}$$

$$A_{10}^{b}=0.015-0.811\theta -0.974\theta^{2}+1.765\theta^{3}$$

$$A_{11}^{b}=-0.585+0.319\theta -0.084\theta^{4}$$

$$A_{12}^{b}=-7.287+53.653\theta -55.081\theta^{2}+0.947\theta^{4}$$

$$A_{13}^{b}=-5.158-77.965\theta +105.085\theta^{2}$$

$$A_{14}^{b}=28.354-41.874\theta^{2}$$

$$A_{2}^{b}=A_{20}^{b}+A_{21}^{b}Y+A_{22}^{b}Y^{2}+A_{23}^{b}Y^{3}+A_{24}^{b}Y^{4}$$

$$A_{20}^{b}=2.501-11.722\theta +14.711\theta^{2}-5.338\theta^{4}$$

$$A_{21}^{b}=20.181-60.484\theta +51.074\theta^{2}-14.228\theta^{3}$$

$$A_{22}^{b}=-15.157-0.689\theta^{2}+35.741\theta^{4}$$

$$A_{23}^{b}=74.171+0.421\theta^{2}-89.665\theta^{4}$$

$$A_{24}^{b}=-108.419+93.296\theta^{2}+1.340\theta^{4}$$

$$A_{3}^{b}=A_{30}^{b}+A_{31}^{b}Y+A_{32}^{b}Y^{2}+A_{33}^{b}Y^{3}+A_{34}^{b}Y^{4}$$

$$A_{30}^{b}=-21.534+82.796\theta -94.723\theta^{2}+18.151\theta^{3}+14.663\theta^{4}$$

$$A_{31}^{b}=-12.022+42.247\theta^{2}-16.989\theta^{4}$$

$$A_{32}^{b}=68.318-111.122\theta^{2}-28.428\theta^{4}$$

$$A_{33}^{b}=-268.940+340.766\theta^{2}+18.190\theta^{4}$$

$$A_{34}^{b}=342.766-505.198\theta^{2}+160.946\theta^{4}$$

$$A_{4}^{b}=A_{40}^{b}+A_{41}^{b}Y+A_{42}^{b}Y^{2}+A_{43}^{b}Y^{3}+A_{44}^{b}Y^{4}$$

$$A_{40}^{b}=30.817-118.209\theta +137.515\theta^{2}-34.910\theta^{3}-14.672\theta^{4}$$

$$A_{41}^{b}=6.060-51.272\theta^{3}+33.481\theta^{4}$$

$$A_{42}^{b}=-188.380+368.847\theta -453.325\theta^{3}+326.318\theta^{4}$$

$$A_{43}^{b}=534.753-856.175\theta +926.225\theta^{3}-645.821\theta^{4}$$

$$A_{44}^{b}=-690.666+1465.07\theta -1261.73\theta^{2}+396.370\theta^{3}+50.486\theta^{4}$$

$$\begin{array}{l} \kappa_{b}=1+\left(\sqrt{Z}-1\right)\left\{1-\left[\left(-1.00+2.23\theta -0.41\theta^{2}\right)+\left(-2.81+37.10\theta -21.04\theta^{2}\right)Y^{2}\right]X\right\}\cdot \\ \;\;\cdot Exp\left\{-{\left[\left(11.77-13.20\theta +5.77\theta^{2}\right)Y\right]}^{2.6}-\left(3.84-4.33\theta +1.68\theta^{2}\right)\right\} \end{array}$$

Shearing load:

(A3)
$$K_{ts}=X^{n_{s}}\left(A_{0}^{s}+A_{1}^{s}X+A_{2}^{s}X^{2}+A_{3}^{s}X^{3}+A_{4}^{s}X^{4}\right)\kappa_{s}$$

where:

$$A_{0}^{s}=1.4361-0.0912\theta^{2}+\left(-0.8777-0.0080\theta^{2}\right)Y^{2}$$

$$A_{1}^{s}=0.1147-0.6461\theta +0.2553\theta^{2}+\left(0.0581+0.1094\theta^{2}\right)Y^{2}$$

$$A_{2}^{s}=-0.5070+0.4287\theta^{2}+\left(0.4582+0.2199\theta^{2}\right)Y^{2}$$

$$A_{3}^{s}=0.7581-0.4544\theta^{2}+\left(-0.7112-0.1743\theta^{2}\right)Y^{2}$$

$$A_{4}^{s}=-0.6625+0.4349\theta^{2}+\left(1.1281-0.5013\theta^{2}\right)Y^{2}$$

$$\begin{array}{l} \kappa_{s}=1+\left(\sqrt{Z}-1\right)\left[1-\left(\left(-0.40+0.67\theta +0.70\theta^{2}\right)+\left(-4.17+18.54\theta -6.94\theta^{2}\right)Y^{2}\right)X^{2}\right]\;\;\cdot \\ \;\;\cdot \;\;Exp\left[-{\left(\left(6.26-5.74\theta +2.52\theta^{2}\right)Y\right)}^{2.0}-\left(3.84-3.31\theta +1.23\theta^{2}\right)\right] \end{array}$$

## Appendix B

### Parametric Formulas for Calculating SCFs, while θ = 45°

Tensile (axial) load:

(A4)
$$K_{tt}=X^{-0.3264}\left(A_{0}^{t}+A_{1}^{t}X+A_{2}^{t}X^{2}+A_{3}^{t}X^{3}+A_{4}^{t}X^{4}\right)\kappa_{t}$$

where:

$$A_{0}^{t}=1.490+0.523Y-8.946Y^{2}+14.813Y^{3}-7.663Y^{4}$$

$$A_{1}^{t}=-0.352-0.177Y+0.872Y^{2}+5.466Y^{3}-5.868Y^{4}$$

$$A_{2}^{t}=0.167+1.0580Y-4.115Y^{2}+13.152Y^{3}-17.032Y^{4}$$

$$A_{3}^{t}=-0.115-3.167Y+10.737Y^{2}-26.319Y^{3}+27.302Y^{4}$$

$$A_{4}^{t}=-0.018+1.742Y-5.021Y^{2}+14.844Y^{3}-16.359Y^{4}$$

$$\kappa_{t}=1+\left(\sqrt{Z}-1\right)[1-(0.568+16.296Y^{2})X]\cdot Exp[-{\left(6.044Y\right)}^{2.4}-1.554]$$

Bending load:

(A5)
$$K_{tb}=X^{-0.3264}\left(A_{0}^{b}+A_{1}^{b}X+A_{2}^{b}X^{2}+A_{3}^{b}X^{3}+A_{4}^{b}X^{4}\right)\kappa_{b}$$

where:

$$A_{0}^{b}=1.487+1.154Y-7.529Y^{2}+6.724Y^{3}-0.075Y^{4}$$

$$A_{1}^{b}=-0.368-0.366Y+1.236Y^{2}-1.570Y^{3}+2.524Y^{4}$$

$$A_{2}^{b}=0.338-2.711Y-1.982Y^{2}+40.313Y^{3}-50.359Y^{4}$$

$$A_{3}^{b}=-0.563+7.574Y-11.045Y^{2}-51.817Y^{3}+92.375Y^{4}$$

$$A_{4}^{b}=0.306-6.040Y+5.853Y^{2}+65.308Y^{3}-107.060Y^{4}$$

$$\kappa_{b}=1+\left(\sqrt{Z}-1)[1-(0.499+13.350Y^{2})X]\cdot Exp[-{\left(4.962Y\right)}^{2.6}-1.476\right]$$

Shearing load:

(A6)
$$K_{ts}=X^{-0.2}\left(A_{0}^{s}+A_{1}^{s}X+A_{2}^{s}X^{2}+A_{3}^{s}X^{3}+A_{4}^{s}X^{4}\right)\kappa_{s}$$

where:

$$A_{0}^{s}=1.380-0.883Y^{2}$$

$$A_{1}^{s}=-0.235+0.126Y^{2}$$

$$A_{2}^{s}=-0.243+0.594Y^{2}$$

$$A_{3}^{s}=0.478-0.819Y^{2}$$

$$A_{4}^{s}=-0.394+0.819Y^{2}$$

$$\kappa_{s}=1+\left(\sqrt{Z}-1)[1-(0.558+6.110Y^{2})X^{2}]\cdot Exp[-{\left(3.306Y\right)}^{2}-1.999\right]$$

## Appendix C

### Alternative Parametric Formulas for Calculating SCF for Plate T-Joints Subjected to Tensile and Bending Loads

Plate T-joint—axial load

Ushirokawa and Nakayama (1983). Range of validity: 0.025 ≤ *ρ*/*t* ≤ 0.35, 20° ≤ *θ* ≤ 50°, *T*/*t* = 1, *h*_p_/*t* = 0.75.

(A7)
$$K_{tt}=1+{\left[\frac{h/\rho }{2.8\left(w/t\right)-2}\right]}^{0.65}\left\{\frac{1-Exp\left[-0.9\theta \sqrt{w/2h}\right]}{1-Exp\left[-0.9\left(\pi /2\right)\sqrt{w/2h}\right]}\right\}$$

$$w=\left(t+2h\right)+0.3\left(T+2h_{p}\right).$$

Tsuji (1990): range of validity not precisely defined.

(A8)
$$K_{tt}=1+1.015{\left[\frac{h/\rho }{2.8\left(w/t\right)-2}\right]}^{0.446}\left\{\frac{1-Exp\left[-0.9\theta \sqrt{w/2h}\right]}{1-Exp\left[-0.9\left(\pi /2\right)\sqrt{w/2h}\right]}\right\}$$

$$w=\left(t+2h\right)+0.3\left(T+2h_{p}\right).$$

Monahan (1995), range of validity: 0.02 ≤ *ρ*/*t* ≤ 0.066, 30° ≤ *θ*≤60°, *L*/*t* = 2.8.

(A9)
$$K_{tt}=1+0.388{\left(\theta \right)}^{0.37}{\left(\rho /t\right)}^{-0.454}$$

Brennan et al. (2000), range of validity: 0.01 ≤ *ρ*/*t* ≤ 0.066, 30° ≤ *θ*≤60°, 0.3 ≤ *L*/*t* ≤ 4.0

(A10)
$$K_{tt}=1.027+0.271{\left(\theta \right)}^{0.216}{\left(\rho /t\right)}^{-0.47}{\left(L/t\right)}^{0.183}$$

Plate T-joint—bending load

Ushirokawa and Nakayama (1983), range of validity: 0.025 ≤ *ρ*/*t* ≤ 0.35, 20° ≤ *θ*≤50°, *T*/*t* = 1, *h*_p_/*t* = 0.75.

(A11)
$$\begin{array}{l} K_{tb}=1+1.9\sqrt{Tanh\left(\frac{2T}{t+2h}+\frac{2\rho }{t}\right)}\left\{Tanh\left[\frac{{\left(2h/t\right)}^{1/4}}{1-\rho /t}\right]\right\}\left[\frac{0.13+0.65{\left(1-\rho /t\right)}^{4}}{{\left(\rho /t\right)}^{1/3}}\right]\cdot \\ \;\;\;\;\;\;\;\;\cdot \left\{\frac{1-Exp\left[-0.9\theta \sqrt{w/2h}\right]}{1-Exp\left[-0.9\left(\pi /2\right)\sqrt{w/2h}\right]}\right\} \end{array}$$

$$w=\left(t+2h\right)+0.3\left(T+2h_{p}\right)$$

Niu and Glinka (1987), range of validity: 0.02 ≤ *ρ*/*t* ≤ 0.066, 30° ≤ *θ*≤60°, *T*/*t* = 1, *h*/*t* = 1.

(A12)
$$K_{tb}=1+0.5121{\left(\theta \right)}^{0.572}{\left(\rho /t\right)}^{-0.469}$$

Tsuji (1990), range of validity not precisely defined.

(A13)
$$K_{tb}=1+\left[0.629+0.058Ln\left(\frac{T+2h_{p}}{t}\right)\right]{\left[\frac{\rho }{t}\right]}^{-0.431}Tanh\left(\frac{6h}{t}\right)\left\{\frac{1-Exp\left[-0.9\theta \sqrt{w/2h}\right]}{1-Exp\left[-0.9\left(\pi /2\right)\sqrt{w/2h}\right]}\right\}$$

$$w=\left(t+2h\right)+0.3\left(T+2h_{p}\right)$$

Brennan et al. (2000), range of validity: 0.01 ≤ *ρ*/*t* ≤ 0.066, 30° ≤ *θ*≤60°, 0.3 ≤ *L*/*t* ≤ 4.0.

(A14)
$$K_{tt}=1.01+0.344{\left(\theta \right)}^{0.336}{\left(\rho /t\right)}^{-0.468}{\left(L/t\right)}^{0.233}$$
